# Corrigendum: *Paenibacillus marinisediminis* sp. nov., a bacterium isolated from marine sediment

**DOI:** 10.71150/jm.2604100

**Published:** 2026-04-07

**Authors:** Hae-Won Lee, Seong Woon Roh, Kyung June Yim, Na-Ri Shin, Jina Lee, Tae Woong Whon, Joon Yong Kim, Dong-Wook Hyun, Daekyung Kim, Jin-Woo Bae

**Affiliations:** 1 Department of Life and Nanopharmaceutical Sciences and Department of Biology, Kyung Hee University, Seoul 130-701, Republic of Korea; 2 Jeju Center, Korea Basic Science Institute, Jeju 690-756, Republic of Korea

Corrigendum: Journal of Microbiology. 2013. 51: 312–317.

https://doi.org/10.1007/s12275-013-3198-2

­

The original online version of this article was revised:

This article contained an error in the geographical coordinates of the sampling site in the Materials and Methods section.

## Materials and Methods

### Bacterial strains

­

**The originally published version read as follows:**

Strain LHW35^T^ was isolated from a marine sediment (34°88′37″N, 127°92′13″E) collected from the bay of Kangjin, Republic of Korea using a dilution-plating technique with pleuropneumonia-like organism agar (PPLOA; Difco, Korea) at 37°C.

­

**It should be read as follows:**

Strain LHW35^T^ was isolated from a marine sediment (34.568483°N, 126.780070°E) collected from the bay of Kangjin, Republic of Korea using a dilution-plating technique with pleuropneumonia-like organism agar (PPLOA; Difco, Korea) at 37°C.

